# Environmental impact of phytobiotic additives on greenhouse gas emission reduction, rumen fermentation manipulation, and performance in ruminants: an updated review

**DOI:** 10.1007/s11356-024-33664-5

**Published:** 2024-05-21

**Authors:** Mariam G. Ahmed, Eman A. Elwakeel, Samir Z. El-Zarkouny, Adham A. Al-Sagheer

**Affiliations:** 1 Agriculture Research Center, Animal Production Research Institute, Nadi El-Said, Giza, 11622 Egypt; 2 Department of Animal and Fish Production, Faculty of Agriculture (El-Shatby), Alexandria University, Alexandria, 21545 Egypt; 3 Animal Production Department, Faculty of Agriculture, Zagazig University, Zagazig, 44511 Egypt

**Keywords:** Phytobiotic additives, Greenhouse gas emissions, Rumen fermentation, Animal health

## Abstract

Ruminal fermentation is a natural process involving beneficial microorganisms that contribute to the production of valuable products and efficient nutrient conversion. However, it also leads to the emission of greenhouse gases, which have detrimental effects on the environment and animal productivity. Phytobiotic additives have emerged as a potential solution to these challenges, offering benefits in terms of rumen fermentation modulation, pollution reduction, and improved animal health and performance. This updated review aims to provide a comprehensive understanding of the specific benefits of phytobiotic additives in ruminant nutrition by summarizing existing studies. Phytobiotic additives, rich in secondary metabolites such as tannins, saponins, alkaloids, and essential oils, have demonstrated biological properties that positively influence rumen fermentation and enhance animal health and productivity. These additives contribute to environmental protection by effectively reducing nitrogen excretion and methane emissions from ruminants. Furthermore, they inhibit microbial respiration and nitrification in soil, thereby minimizing nitrous oxide emissions. In addition to their environmental impact, phytobiotic additives improve rumen manipulation, leading to increased ruminant productivity and improved quality of animal products. Their multifaceted properties, including anthelmintic, antioxidant, antimicrobial, and immunomodulatory effects, further contribute to the health and well-being of both animals and humans. The potential synergistic effects of combining phytobiotic additives with probiotics are also explored, highlighting the need for further research in this area. In conclusion, phytobiotic additives show great promise as sustainable and effective solutions for improving ruminant nutrition and addressing environmental challenges.

## Introduction

Greenhouse gases (GHGs) such as carbon dioxide (CO_2_), methane (CH_4_), nitrous oxide (N_2_O), and fluorinated gases accumulate in the atmosphere and cause global warming, which influences climate change and poses serious environmental risks (Zandalinas et al. 2021). Approximately 14.5% of global GHG emissions are attributable to the livestock industry (Kristiansen et al. 2021). More than 90% of the CH_4_ emissions from livestock and 40% of the agricultural GHG emissions are generated by the enteric fermentation process (Tubiello et al. 2013). Additionally, ruminant GHG emissions have a detrimental effect on the economy because they reduce energy availability for ruminant productivity (Bekele et al. 2022).

To achieve better fermentative efficiency, manipulating rumen fermentation has become an important area of research in animal nutrition to reduce GHG emissions, improve nutrient utilization, and enhance animal performance (Gislon et al. 2020). Because forage alone cannot support high levels of animal productivity and rumen fermentation manipulation, feed additives must be used to optimize rumen function by changing the composition and activity of the microbial population in the rumen (Almeida et al. 2021).

Ionophores are effective feed additives in ruminant diets that reduce nitrogen (N) excretion and CH_4_ emissions into the environment, improve animal productivity, and modify rumen fermentation (Marques and Cooke 2021). However, the use of ionophores has been banned in the EU (Directive 1831/2003/EC) due to the potential passage of residues into milk and the increased risk of the emergence of multi-drug-resistant bacteria in human health (Abadi et al. 2019; Ayyat et al. 2021). As a result, most recent investigations have focused on using natural alternatives for antibiotics, such as phytobiotic additives.

Phytobiotic additives contain a high concentration of natural bioactive components, which have a variety of activities such as antioxidant, anthelmintic, anti-inflammatory, and immunostimulant properties, as well as antimicrobial activity against some pathogenic organisms and promote the proliferation and growth of beneficial bacteria in the gut (Alsaht et al. 2014; Sharma et al. 2022). In light of this, the main objective of this review is to discuss recent findings on the potential benefits of phytobiotic additives in terms of environmental impact, rumen fermentation, animal performance, and product quality, as well as their effects on animal health status. Additionally, we discuss the synergistic effect of phytobiotic additives and other feed additives.

## Overview of phytobiotic additives and their use in ruminant nutrition

**Fig. 1:**
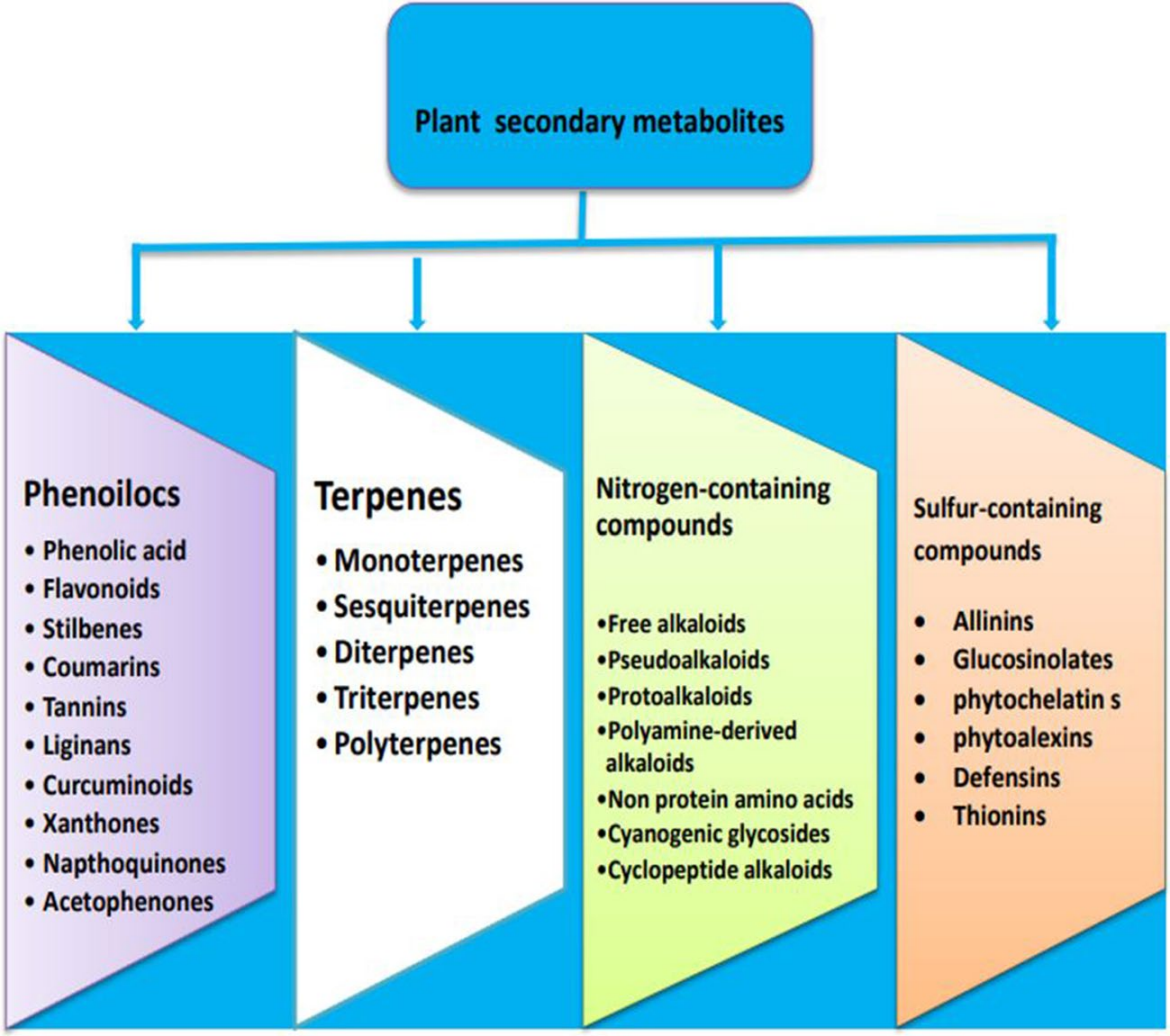
Classification of plant secondary metabolites

Phytobiotic additives are rich plant secondary metabolites (PSMs), organic substances known as phytochemicals, phytobiotics, or herbal and botanical compounds found in plant tissues that form byproducts of various emergency metabolic processes that occur in various plant tissues (Franz et al. 2020). Secondary metabolites are found in legume trees, medicinal plants, and spices, as well as agricultural byproducts from industrial processing (García-Ruiz et al. 2012). There are approximately 100,000 PSMs synthesized in plants. These compounds are similar in many vital activities, metabolic behaviors, and many natural and chemical properties. They can be classified into four major classes: phenolics, terpenes, nitrogen-containing compounds, and sulfur-containing compounds (Sharma et al. 2022), as shown in Fig. 1. In recent years, research has focused on the use of phytobiotic additives as an alternative for ionophores in ruminant diets, thereby avoiding toxic residues in products (e.g., milk and meat) and microbial resistance, thus rendering it safe for both animals and humans (Abadi et al. 2019). Phytobiotic additives in ruminant diets can take the following forms: (i) Herbs are solid, dry, or ground or extracts (crude, concentrated, or dry) (Franz et al. 2020). (ii) Essential oils are volatile plant compounds extracted from plant material via steam distillation using either water or aqueous alcohol (O’Bryan et al. 2015). (iii) Oleoresins are semi-solid extracts containing resin and essential or fatty oils extracted from spices using a hydrocarbon solvent (An et al. 2023). Previous research has shown that phytobiotic additives have a positive effect on animal nutrition, as shown in Table 1. Phytobiotic additives are used as flavor enhancers due to their odor properties, and they also modify rumen fermentation and the microbial population in the rumen due to their antimicrobial activity (Ricci et al. 2021).

**Table 1:** The effects of phytobiotic additives on rumen fermentation, animal performance, and health status

Phytobiotic additives	Secondary compounds	Form used	Species animals	Dose	Effects	Reference
*Acacia mearnsii*	Condensed tannins	Extract	Lamb	0, 20, 40, 60, and 80 g CT/kg dry matter (DM)	-Improve quadratically intake of nutrients -Increase average daily gain and N retention	Costa et al. (2021)
			Dairy cow	30 g acacia extract/kg of dietary DM	-Reduce methane emission -Modified milk fatty acids	Denninger et al. (2020)
		Fresh	Lamb	0, 50, 100, 150, and 200 g/kg DM	-Improve the meat’s beneficial FA profile -Enhance meat antioxidant capacity, tenderness, and lipid shelf-life	Uushona et al. (2023a)
*Caragana korshinskii Kom*	Phenolic acids Alkaloids	Fresh	Sheep	10% of the diet forage	-Improve fiber digestion -Enhance feed conversion efficiency	Wang et al. (2022b)
*Eucalyptus citriodora* *Populus deltoides*	Essential oils, flavonoids, condensed tannins, volatile isoprene	Dried mixture	Buffaloes	15 g kg^−1^ feed dry matter	-Improve milk production and n-cell-mediated and humoral immune response -Increase nutrient digestibility -Mitigate enteric methane	Dey et al. (2021)
			Buffalo calves	1.3% or 3.9% of feed dry matter intake	-Improve the immune response and antioxidant status -Reduce enteric methane emission	Kumar et al. (2022)
*Capsicum*	Capsaicin	Extract Oleoresin	Buffaloes	0, 10, 20, or 40 mg/kg of dry matter	-Enhance milk production and chemical composition of milk -Increase quadratically DM intake -Reduce rectal temperature and respiratory rates	An et al. (2023)
*Citrus reticulata*	Flavonoids	Extract	Dairy cow	50, 100, and 150 g/h/d	-Improved immune-metabolic status -Reduce inflammatory response -Modulated sphingolipid metabolism and secondary bile acid production in the hindgut	Zhao et al. (2023)
*Onobrychis viciifolia*	Condensed tannins Flavonoid	Ensiled	Dairy cows	Replacing 50% of grass silage	-Decrease ruminal biohydrogenation -Enhance milk content of vaccenic acid and PUFA	Huyen et al. (2020)
		Fresh	Suckling lambs	Ad libitum with a supplement of barley (200 g/d)	-Reducing excreted coccidian oocysts -Enhance antioxidant-immune status	Pelegrin-Valls et al. (2022)
*Gliricidia sepium* *Enterolobium cyclocarpum*	Tannin and saponin	Dried	Heifers	0, 15, 30, and 45% DM basis	-Reduce methane emission per unit product at low concentration -No effect on rumen microbial population or daily dry matter intake	Molina-Botero et al. (2019)
*Vitis vinifera*	Hydrolyzable tannins Condensed tannins	Dried	Dairy cow	13% of DM	-Reduces methane yield -No effect on urine N excretion -Low n-6:n-3 ratio and high vaccenic and rumenic acids	Birkinshaw et al. (2022)
*Hedysarum flexuosum L*	Tannins	Hay	Goat	35 or 70% on a DM basis	-Enhance the antioxidant capacity -Improve the milk’s beneficial FA profile, such as total monounsaturated, polyunsaturated	Boukrouh et al. (2023)
*Arnica montana*	2-Hexanal, myrcene, limonene, glycerol tricaprylate	Extract (essential oil)	Lamb	0, 450, 900, and 1350 mg/kg DM	-Increased weight gain -Enhance carcass characteristics and the fatty acid profile of the meat	Dias Junior et al. (2023)
*Phyllanthus emblica*	Phenolic acids, flavonoids, tannin, and alkaloids	Fresh	Dairy Cows	200/400/600 g/d	-Improved antioxidant capacity -Enhance protein efficiency -Improve the milk’s beneficial FA profile	Tilahun et al. (2022)
*Herbal tea residue*	Flavones, alkaloids, and volatile oils	Ensiled	Heifers	5 and 10% of DM	-Improve growth performance -Enhance energy utilization efficiency -Improve immunity and antioxidant function -Alleviated heat stress	Xie et al. (2020)
*Broussonetia papyrifera* L	Flavonoids Terpene	Ensiled	Dairy cows	5, 10, and 15% of DM	-Low milk somatic cell count -Increase the PUFA content of milk -Raise immune and antioxidant functions	Si et al. (2018)
			Sheep	15, 30, and 45% of DM	-Enhance growth performance -Increase the content of n-3 PUFA in the *longissimus dorsi* muscle -Improve immunity and antioxidant capacity	Si et al. (2019)
		Hay	Lamb	30, 60, and 100% of DM	-Improve average weight gain and average daily gain -Enhance meat quality and immune performance	Sheng et al. (2021)
*Morus alba*	Flavones, flavonols, and other polyphenols	Extract	Buffaloes	15, 30, and 40 g/h/d	-Improve milk yield and chemical composition -Improve the concentration of serum metabolic hormones -Reduce heat-induced oxidative stress during the summer season	Li et al. (2020)
*Leucaena leucocephala*	Condensed tannin	Fresh	Heifers	0%, 20%, 40%, 60%, and 80% of dry matter	-Reduce enteric methane emissions without affecting dry matter intake and protozoa population at level 80%	Piñeiro-Vázquez et al. (2018)
*Moringa oleifera*	Polyphenols	Ensiled	Dairy cows	180 g/kg TMR DM	-Increase milk production and energy-corrected milk -Enhance the antioxidative activity of milk to more than 20%	Cohen-Zinder et al. (2016)
Curcumin	–	Extract	*Lambs*	0, 300, 600, and 900 mg/kg	-Promote rumen fermentation and growth performance -Improve rumen microbial activity -Enhance microbial protein synthesis -Enhance serum antioxidant activity	Tian et al. (2023)
Grape seed	Flavanols	Extract	Preweaning dairy calves	25, 50, and 100 mg/kg BW/d	Enhance antioxidant activity, inflammation, and hematological parameters and reduce respiratory rate under heat stress	Urkmez and Biricik (2022)

Phytobiotic additives are used to improve livestock productivity, product quality, and health status due to their antioxidant, antimicrobial, anthelmintic, anti-inflammatory, and immunostimulant properties (Sharma et al. 2022). Additionally, some phytobiotic additives can reduce environmental pollutants caused by ruminant N excretion and CH_4_ emissions (Gao et al. 2022; Montoya-Flores et al. 2020).

## Mechanisms of action and catabolism of phytobiotic additives in the rumen

The rumen is a large fermentation chamber located in ruminant animals’ digestive systems that is home to billions of microorganisms such as bacteria, protozoa, and fungi. These microorganisms break down plant material into microbial biomass and fermentation end products that can be utilized by the host animal (Owens and Basalan 2016). Phytobiotic additives have been proposed as good candidates for modifying the population of specific bacteria groups in the rumen to maximize energy and protein utilization (Dey et al. 2021; Tian et al. 2023). Phytobiotic additives have been shown to act in the rumen similarly to antibiotics, with strong broad-spectrum effects against microorganisms, including Gram-positive and Gram-negative bacteria (Dias Junior et al. 2023). Phytobiotic additives’ antimicrobial activity may be attributed to the hydrophobicity of PSCs, which may influence microbial cell surface properties such as electron transport, ion gradients, protein translocation, and enzyme-dependent reactions, all of which cause induced changes in bacterial morphology, reduced nutrient transport into the cell, and decreased bacterial growth (McSweeney et al. 2001; Smith et al. 2005). The effect of phytobiotic additives on ruminal microbe activity is dependent on the dose, type, and chemical profiles of the compounds in plants (López et al. 2010), as well as differences between compounds within each class of compound on rumen bacteria, as demonstrated by Seradj et al. (2016), who found that there was variation between flavonoid substances on lactic acid producer *S. bovis*, which was significantly decreased with neohesperidine, poncirine, and isonaringine and significantly increased with neoeriocitrine compared to control. Furthermore, the molecular weight of phytobiotic compounds was linked to ruminal microbes; tannins with a low molecular weight inhibit rumen microbes more effectively (Patra and Saxena 2011). The procyanidin/prodelphinidin (PC/PD) ratio, degree of polymerization, and cis/trans ratio are important factors that influence the impact of phytobiotic compounds, such as condensed tannins (CTs), on ruminal microbe activity. The PC/PD ratio can affect the biological activity of CTs, as demonstrated in sainfoin (*Onobrychis viciifolia*) (Hatew et al. 2016). The degree of polymerization and cis/trans ratio also play a role in the biological activity of CTs, as shown by a negative correlation between nitrogen solubility and these factors in sainfoin (Lagrange et al. 2021).

Phytobiotic compounds, especially phenolic compounds, were reported to be absorbed directly through the rumen wall and entered the circulatory system, exhibiting antioxidant effects on host animals and friendly environmental conditions, while others were partially catabolized by rumen microbes via catabolic pathways (Bao et al. 2018; Kim et al. 2021). For instance, Kim et al. (2021) observed that hydroxycinnamic acids such as coumaric acid, ferulic acid, and caffeic acid decreased after 12 h of in vitro incubation in rumen fluid and were more than 70% decreased at 72 h. This reduction is explained by the ruminal degradation of hydroxycinnamic acids by rumen microbes via reduction, demethylation, dihydroxylation, or decarboxylation pathways into natural products, which are commercial and environmentally friendly renewable energy sources, as reviewed by Wang et al. (2022c).

Flavonoid ring systems (e.g., quercetin and kaempferol) and phenolic glycosides such as rutin and naringin could be partially hydrolyzed by rumen microbiota by hydroxylic ring cleavage into acetate, butyrate, 3,4-dihydroxyphenylacetic acid, phloroglucinol, and 4-methylcatechol during in vitro inoculum (Berger et al. 2015) and then likely absorbed in the small intestine (Gohlke et al. 2013). The mechanisms involved in the transport of flavonoids in the rumen and intestines are not well understood. However, Murota and Terao (2003) provided an overview of the proposed mechanisms for the absorption and transport of quercetin glucosides in the intestines. The mechanism of quercetin absorption in the intestine involves several steps. Firstly, it needs to be solubilized by bile salts and other emulsifying agents present in the gut lumen. This solubilization allows for better interaction with the absorptive surfaces of the intestine. Once solubilized, quercetin can pass through the intestinal epithelial cells via two main pathways: passive diffusion and active transport. Passive diffusion occurs when quercetin glucosides can be broken down by lactase phloridzin hydrolase, an enzyme found in the brush border membrane, resulting in aglycones that can be absorbed through lipophilic simple diffusion. Active transport mechanisms also play a role in quercetin monoglucoside absorption, but they are then cleaved by cytosolic β-glucosidase hydrolysis. These mechanisms involve carrier proteins located on the surface of intestinal cells that actively transport quercetin molecules from one side of the cell membrane to another against their concentration gradient. Once inside intestinal cells, quercetin aglycones can undergo further metabolism, where the compound can be conjugated with various molecules, such as glucuronic acid by UDP-glucuronosyltransferase or sulfate by phenol sulfotransferase, to form water-soluble metabolites. These metabolites are then transported out of the cells and into the bloodstream.

Additionally, rumen microbes such as *Selenomonas ruminantium* and *Streptococcus* spp. can break down hydrolyzable tannins (HT) by producing esterase and tannin acylhydrolase to generate gallic acid and ellagic acid (Goel et al. 2005). The gallic acid in beef cattle is decarboxylated in the rumen to pyrogallol, which is then converted into resorcinol as urinary metabolites with their respective inhibitive effects on decreasing urine N_2_O-N emissions (Bao et al. 2018; Zhou et al. 2019).

Condensed tannins have the potential to bring about positive changes in the rumen through various mechanisms. They can regulate proteolysis during forage preservation and ruminal digestion, prevent bloat, decrease intestinal parasite burdens, and mitigate methane and ammonia emissions from ruminants (Zeller 2019). By incorporating CTs into the animal feed at appropriate levels, ruminants can optimize protein utilization and minimize losses due to excessive protein breakdown in the rumen. It is crucial to strike a balance so that the added CTs do not adversely impact microbial protein synthesis in the rumen. Furthermore, the amount of CT supplementation should be carefully controlled to ensure it remains within safe limits for animal consumption, avoiding any potential toxic effects (Besharati et al. 2022).

On the other hand, the degradation of CTs in rumen fluid remains unclear. An earlier study found that rumen microbes are unable to degrade CTs due to a lack of enzymes and the fact that their phenolic hydroxyl groups are combined with other macromolecules (Naumann et al. 2017). Notwithstanding, Rira et al. (2022) investigated the relationship between the disappearance of the free and bound CT fractions of tropical tannin-rich plants in vitro and in situ. The findings showed that free CT from all plants completely disappeared after a 24-h incubation in the rumen. Condensed tannins that were protein-bound disappeared at varying rates, from 93% in *Gliricidia sepium* to 21% in *Acacia nilotica*. Contrarily, the disappearance of CTs bound to fiber averaged 82% and was consistent across all plants. More research is needed to get a better understanding of the microbial degradation of CTs.

## Influence of phytobiotic additives in mitigating the environmental impact of ruminant production

Global warming is caused by the accumulation of GHGs in the atmosphere, specifically CO_2_, CH_4_, N_2_O, and fluorinated gases (e.g., hydrofluorocarbons, perfluorocarbons, sulfur hexafluoride, and nitrogen trifluoride) (Zandalinas et al. 2021). The infrared radiation emitted by the planet’s surface after sunlight has been absorbed is what is causing the alarming trend of an ongoing rise in ocean and surface temperatures (Zandalinas et al. 2021). According to the intergovernmental panel on climate change (IPCC 2019), the average land surface air temperature increased by 1.53 °C (1.38–1.68 °C) between 1850 and 2015, while the average global surface temperature (land and ocean) increased by 0.87 °C (0.75–0.99 °C). Climate change brought about by global warming has resulted in increased rainfall intensity, flooding, drought frequency and severity, heat stress, dry spells, wind, sea-level rise, wave action, and permafrost thaw, with the effects being influenced by land management (IPCC 2019).

**Fig. 2:**
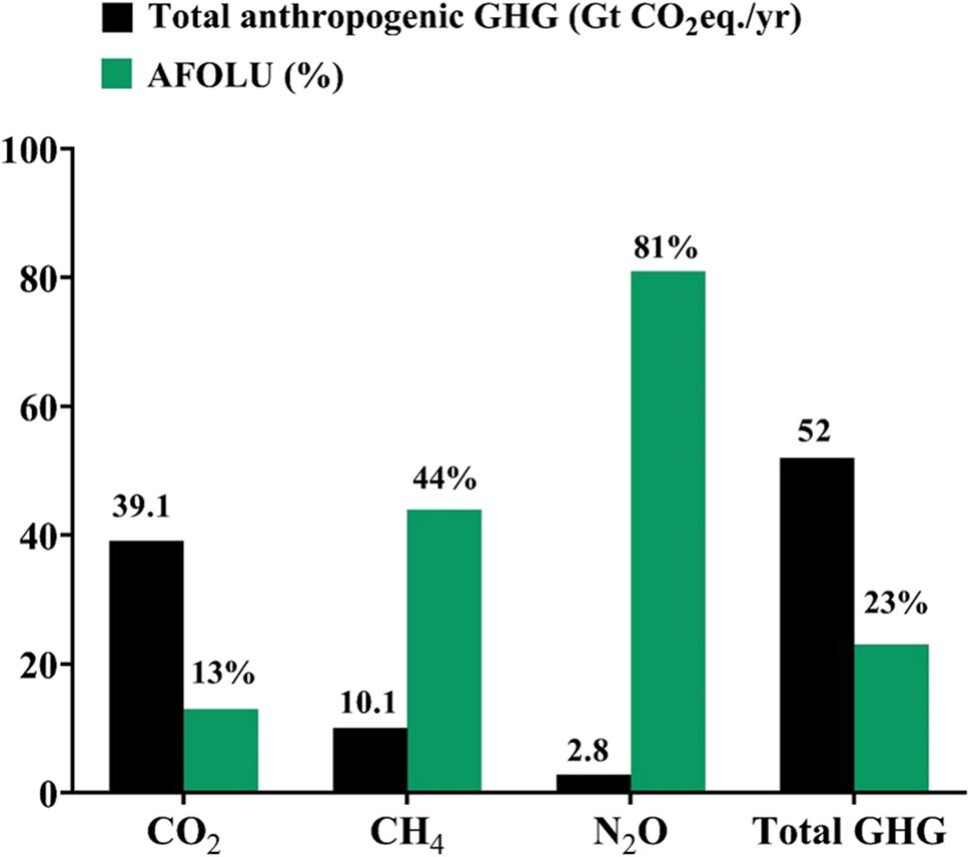
Total anthropogenic greenhouse gas (GHG) emissions in gigatons of CO_2_ equivalent per year (Gt CO_2_ eq./year) and the proportion of anthropogenic GHG emissions from agriculture, forestry, and other land use (AFOLU; average for 2007–2016) according to the Intergovernmental Panel on Climate Change (IPCC 2019)

**Fig. 3:**
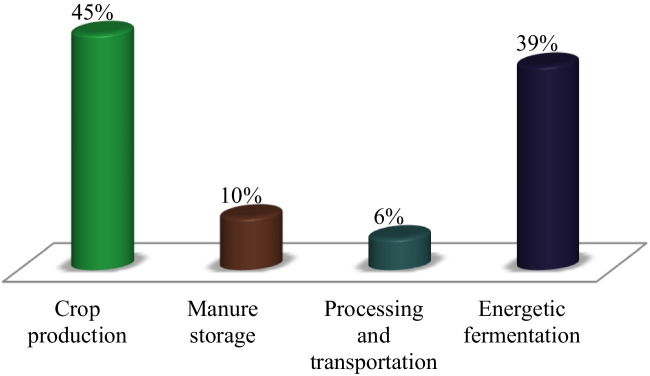
Greenhouse gas emissions derived from crop production, manure storage, processing and transportation and energetic fermentation, according to Gerber et al. (2013)

Around 13% of CO_2_, 44% of CH_4_, and 81% of N_2_O emissions from human activities worldwide between 2007 and 2016 were attributed to agriculture, forestry, and other land use activities, making up 23% of all net anthropogenic GHG emissions (IPCC 2019) as shown (Fig. 2). Crop production and enteric fermentation produce the most GHGs, accounting for 45 and 39% of total sector emissions, respectively, while manure storage and processing and animal product transportation contribute 10 and 6%, respectively (Gerber et al. 2013) (Fig. 3). The livestock industry contributes significantly to GHG emissions, accounting for 14.5% of global emissions (Kristiansen et al. 2021).

Approximately 80 to 95 million tonnes of CH_4_ are predicted to be released annually into the atmosphere by ruminant livestock, as reviewed by Bačėninaitė et al. (2022). Methane forms as a byproduct of this fermentative process when hydrogen (H_2_) and CO_2_ are released and used by methanogenic archaea (Boadi et al. 2004). Methane is primarily expelled from the rumen through eructation and absorbed into the blood system (Boadi et al. 2004), but it is also emitted from manure storage (Gerber et al. 2013). According to the latest evaluation by the National Oceanic and Atmospheric Administration (NOAA 2022), the atmospheric CH_4_ concentration has set another record in 2021. The report states that the global average atmospheric methane concentration reached a new high of 1895.7 parts per billion (ppb) in August 2021, which is an increase of 15 ppb from the previous year. Based on data from NOAA (2022), scientists estimate that global methane emissions were 15% higher in 2021 compared to the period between 1984 and 2006. Methane is a potent greenhouse gas with a warming potential that is more than 28 times greater than CO_2_ (IPCC 2013). The increase in atmospheric CH_4_ concentration is, therefore, a cause for concern as it contributes significantly to global warming and also has a negative impact on the economy as it can lead to a decrease in energy availability for ruminant animals and lower feed utilization efficiency (Bekele et al. 2022).

Nitrous oxide is also a powerful greenhouse gas, with a warming potential of over 265 times that of CO_2_ (IPCC 2013). In livestock production, 70–80% of dietary protein is hydrolyzed to ammonia (NH_3_) in the rumen, and a low protein ratio leaves the rumen undegraded (Hristov et al. 2011). When the rumen produces too much NH_3_, it absorbs N from the rumen wall, converts it to urea in the liver, and is then eliminated in the urine by the kidney, resulting in N loss (Gao et al. 2022). The amount of nitrogen to be excreted by the kidney is closely related to the protein balance (quantity and type) and energy offered to the ruminant in the diet, among other factors such as passage rate and metabolism according to growth or production stage. An unbalanced diet with a higher protein content will promote a higher release of N (Chadwick et al. 2018). Meanwhile, when urea is released into the environment, microbial urease converts it to NH_3_, of which a large portion is converted to ammonium (NH_4_), and any remaining NH_3_ quickly undergoes simple chemical reactions, primarily with atmospheric acids such as sulfuric and nitric acid, to form ammonium sulfate, ammonium bisulfate, or ammonium nitrate, all of which are harmful to human health and contribute to environmental pollution (Hristov et al. 2011). On the other hand, NH_4_^+^ in excreta and soil is converted into N_2_O by microbial processes of nitrification and denitrification, which contribute to global warming (Gao and Zhao 2022).

### Effects of phytobiotic additives on methane emission

Several studies have reported that phytobiotic additives are used as antimethanogenesis agents in the rumen, which is thought to directly inhibit the population of methanogens and microbes that produce hydrogen, lowering CH_4_ emissions (Alayón-Gamboa et al. 2023; Chen et al. 2021). According to Rira et al. (2022), archaea diversity decreased in high-tannin-containing *Calliandra calothyrsus* and *Acacia nilotica* at 12 h of incubation. Furthermore, using phytobiotic plants in ensiling form, such as *Neolamarckia cadamba and* grape pomace, reduced the relative abundance of *Methanobrevibacter*, particularly *Methanobrevibacter curvatus*, *Ruminococcaceae NK4A214*, *Ruminococcaceae UCG-010*, and *Christensenellaceae R-7*, while increasing the abundance of *Succiniclasticum* (Zhang et al. 2022; Zhou et al. 2021). The bioactive components of these plants may explain their inhibition effect on methanogenic bacteria; Al-Sagheer et al. (2018) observed a negative linear correlation between CT and CH_4_ production in vitro when guava leaves were used instead of berseem hay containing 1.60, 2.40, 3.14, and 3.10 g CT/kg DM. Moreover, it has been reported that phytobiotic additives do not affect methanogens, although CH_4_ production decreases (Chen et al. 2021; Wang et al. 2022a). In reality, there is a more indirect pathway to reduce CH_4_ production. For example, using microbiota and Pearson correlation analysis data, it was demonstrated that using tannin acid as a feed additive in alfalfa silage reduced rumen CH_4_ emissions by inhibiting protozoa, anaerobic fungi, and cellulolytic bacteria rather than methanogens (Chen et al. 2021), because these microbiotas are H_2_ suppliers as well as biosynthesis of acetate, butyrate for methanogenesis (Abarghuei and Salem 2021). On another pathway, phytobiotic additives increased the relative abundance of propionate-producing species such as *Succiniclasticum* (Zhang et al. 2022). The propionate-producing bacteria can compete with methanogenic bacteria for H_2_ in the rumen, which consequently decreases CH_4_ production (Boadi et al. 2004).

Additionally, several studies found that certain phytobiotic additives had specific effects on CH_4_ production. For instance, according to Fagundes et al. (2020), tannin-rich forages such as *Flemingia macrophylla*, *Leucaena leucocephala*, *Stylosanthes guianensis*, *Gliricidia sepium*, *Cratylia argentea*, *Cajanus cajan*, *Desmodium ovalifolium*, *Macrotyloma axillare*, *Desmodium paniculatum*, and *Lespedeza procumbens* mitigated enteric CH_4_ in vitro, and the lowest CH_4_ production was observed with *Leucaena leucocephala*. Furthermore, Aragadvay-Yungán et al. (2022) evaluated different tropical forage legumes, including *Clitoria arborea*, *Erythrina fusca*, *Bauhinia forficata*, *Erythrina poeppigiana*, *Cratylia argentea*, *Gliricidia sepium*, *Cassia tora*, and *Flemingia macrophylla*. The results indicated that the lowest CH_4_ production was found with *C. arborea*. On the other hand, some studies have shown that phytobiotic additives such as oak tannin extracts (Focant et al. 2019) and artichoke bract silage (Ahmed et al. 2023) had no significant effect on enteric CH_4_ production in vivo and in vitro, respectively.

Differences in CH_4_ production responses of phytobiotic additives in various research could be attributed to several factors, including (i) dietary composition (Ahmed et al. 2021) and forages (e.g., type of forage, chemical composition, and maturity) as well as the quality of the fermentation process during silage-making if phytobiotic additives are ensiled (Evans 2018), (ii) phytobiotic compound type and concentration in the diet, which has been reported that diets with a high HTto CT ratio reduce CH_4_ emissions (Bhatt et al. 2023; Rira et al. 2019), (iii) animal species also have an impact on CH_4_ emissions; Alvarado-Ramírez et al. (2023) found that co-ensiling maize with *Moringa oleifera* reduced CH_4_ (mL g^−1^ DM degraded) in steers when compared to sheep as inoculum sources.

### Effects of phytobiotic additives on nitrous oxide emission

Phytobiotic additives have been shown to be effective at reducing N excretion and thus N_2_O emissions. This is due to the presence of PSMs such as tannin, anthocyanins, aucubin, and glucosinolates (Gao and Zhao 2022; Lazzari et al. 2023). Plantain leaf extract and aucubin solution, when applied to a ruminant urine patch, reduced N_2_O by 50% and 70%, respectively, according to Gardiner et al. (2018). The inhibition effect of phytobiotic additives on reducing urine N_2_O emission can be explained by the shift of N excretions from urine to feces in beef cattle; Gao et al. (2022) evaluated different levels of rapeseed cake containing high glucosinolates at four levels (0, 2.7, 5.4, and 8.0% DM) in the diet of steers. The study’s results showed an increase in the ratio of fecal N to urinary N, linearly increased the urinary excretions of allantoin and the total urinary purine derivatives, and decreased the urea-N-to-urinary N ratio. Uushona et al. (2023b) found that adding *Acacia mearnsii* leaf meal up to 100 g/kg DM in lamb increases fecal N and decreases urine N because fecal N_2_O emissions are much lower than urinary N_2_O emissions (de Klein and Ledgard 2005). Recently, studies on tannic acid as a source of HT revealed that the ratio of hippuric acid-N to urinary N increased and decreased urine N_2_O-N emissions (Zhou et al. 2019). Hippuric acid is thought to be an inhibitor of the soil nitrification process, reducing N_2_O formation. Furthermore, the hydrolysis of PSMs by microbial enzymes in the digestive tract of animals into bioactive products may contribute to the inhibition of urine N_2_O emission (Gao and Zhao 2022). For instance, thiocyanates, metabolites of glucosinolates that have an inhibitory effect on microbial respiration and nitrification in soil, were found to be higher in the plasma and urine of steers fed rapeseed cakes high in glucosinolates (Gao et al. 2022).

## Effects of phytobiotic additives on rumen fermentation

### Effects on ruminal pH

Although the rumen pH allows variations ranging from 5.5 to 7.0, it still requires regulation (Owens and Basalan 2016). The pH level in the rumen is influenced by several factors, including nutritional (e.g., diet composition, feeding frequency, feed intake, and saliva production) and environmental (e.g., heat stress) (Sales et al. 2021). The regulation of rumen pH is essential for maintaining a healthy rumen environment and optimal microbial activity, thereby promoting overall animal health and performance (Owens and Basalan 2016).

Phytobiotic additives have been shown to be effective in maintaining ruminal pH and improving ruminal fermentation, particularly in high-grain production (Rivera-Chacon et al. 2022). For example, using phytobiotic additives such as plant-derived alkaloids (Mickdam et al. 2016), β-sitosterol (Xia et al. 2020), tannic acid (Zhao et al. 2021), and phenolic plant extracts (Ahmed et al. 2022) inhibits the growth of lactate-producing bacteria and promotes the growth of lactic acid utilization bacteria, thereby increasing ruminal pH and reducing lactate concentration and lipopolysaccharide accumulation.

Recently, it has been suggested that botanical compounds (e.g., essential oils) may stimulate salivation due to their smell properties as well as improve the physicochemical composition of the salivary and salivary proteome, which are linked to rumen function, host metabolism, and immune response in animals fed a high-concentrate diet (Castillo-Lopez et al. 2023; Ricci et al. 2021). According to Ricci et al. (2021), analysis of stimulated saliva revealed that garlic oil and ginger increased phosphate concentration, while thyme oil increased osmolality and capsaicin and thymol increased buffer capacity. Although some research has shown that phytobiotic additives have a selective effect on lactate-producing bacteria (Ahmed et al. 2022; Seradj et al. 2016), this mechanism could be explained by the types and concentrations of active components as well as the antagonistic effect of active components on rumen bacteria (Hajimehdipoor et al. 2014; Seradj et al. 2016) and bacterial resistance to active components (Kim et al. 2021).

### Effects on ruminal volatile fatty acids

Ruminal volatile fatty acids (VFA) produced during microbial fermentation of plant materials meet the majority of ruminant energy requirements (Owens and Basalan 2016). The effects of phytobiotic additives on VFA production range from no change (Khurana et al. 2023; Safari et al. 2018) to increased production (Tian et al. 2023; Yaxing et al. 2022) to inhibition of VFA production (Della Rosa et al. 2022; Pech-Cervantes et al. 2021). Furthermore, numerous studies (Khurana et al. 2023; Ma et al. 2020; Orzuna-Orzuna et al. 2022) have shown that phytobiotic additives, either plant extracts or active components—shift VFA molar proportions like monensin (i.e., decrease acetate and increase propionate). Propionate serves as an energy source for some anabolic functions in ruminants, so it is hypothesized that phytobiotic additives improve the utilization of energy to achieve better productivity performance (Chen et al. 2020). Meanwhile, propionate serves as the main alternative H^+^ sink and consequently reduces methane production (Wang et al. 2018). The increase in propionate proportions caused by phytobiotic additives could be explained by altering the ruminal bacterial community, such as Succinivibrionaceae, which was increased by garlic and citrus extract (Khurana et al. 2023) and ensiling grape pomace (Zhang et al. 2022). This bacterium is associated with improved feed efficiency, lower CH_4_ emissions, and higher propionate concentrations due to competition with hydrogenotrophic methanogens for substrate and propionate produced via the succinate pathway (Ramayo-Caldas et al. 2020). Some studies, on the other hand, found that phytobiotic additives such as curcumin (Tian et al. 2023), *Allium mongolicum* Regel essential oil (Yaxing et al. 2022), and citrus flavonoid extracts (Zhao et al. 2023) increased ruminal acetate concentration, which is associated with improved fibrinolytic bacteria and enzyme activities in the rumen with strong fiber degradation ability (Yaxing et al. 2022).

### Effects on ammonia concentration and microbial protein synthesis

Likewise, there are significant benefits of phytobiotic additives in the rumen, such as reduced protein degradation to NH_3_, which increases escape protein to the duodenum, as well as improved efficiency of microbial protein synthesis and bacterial N flow from the rumen, which is the main supplier of amino acids for ruminants and is critical to animal performance (Abarghuei and Salem 2021, Abd’quadri-Abojukoro and Nsahlai 2023, Al-Sagheer et al. 2018).

Various mechanisms could explain the reduction of NH_3_ concentrations and enhance microbial protein synthesis in the rumen by phytobiotic additives. As an illustration, tannin forms a complex with protein via hydrogen bonding, and its hydrophobic nature protects it from hydrolysis by rumen microbial enzymes (Mueller-Harvey et al. 2019), as well as tannins’ inhibitory effects on proteolytic bacteria (Abarghuei and Salem 2021). Furthermore, secondary metabolites such as saponin and tannins may have antiprotozoal properties that contribute to NH_3_ reduction, which is likely due to decreased bacterial lysosome activity or an increase in NH_3_-N uptake for microbial protein biomass synthesis (Abarghuei and Salem 2021; Kholif 2023; Tian et al. 2023).

## Influence of phytobiotic additives on animal performance

Several studies have shown that phytobiotic additives enhance growth performance and increase the efficiency of ruminant milk or meat production, as outlined in Table 1. Phytobiotic additives such as essential oils (Dorantes-Iturbide et al. 2022; Yaxing et al. 2022) and high tannin-containing forage (Wang et al. 2022b; Xie et al. 2020) improved dry matter intake (DMI), nutrient digestibility, average daily gain (ADG), and feed conversion ratio in beef production.

However, there are limits to the responses of phytobiotic additives to growth performance, as demonstrated by Dezah et al. (2021) that replacing Glycine max with *Acacia mearnsii* at 500 g/kg DM of diet steers reduced DMI, ADG, feed efficiency, and carcass weights. Avila et al. (2020) observed that CT extracts from black wattle (*A. mearnsii*) did not affect nutrient utilization in steers fed 0, 5, 10, 15, and 20 g/kg diet DM. In contrast, Costa et al. (2021) found that including CT from *A. mearnsii* extract up to 40 g CT/kg dietary DM improves DMI and ADG, thereby improving utilization efficacy in lambs. A variety of factors contribute to this, including their level of inclusion in the diet, the type and concentration of PSCs in plants (Pech-Cervantes et al. 2021), and the physiological status of the consuming species (Benchaar et al. 2008).

Furthermore, phytobiotic additives have been shown to improve lactation performance by increasing energy-corrected milk yield and milk composition of fat, protein, and total solids and improving feed utilization efficiency for milk production, such as citrus flavonoids extracts (Zhao et al. 2023), *Capsicum oleoresin* (An et al. 2023), high tannin-containing forage (Dey et al. 2021; Gannuscio et al. 2022), and essential oils (Kalaitsidis et al. 2021; Silvestre et al. 2022).

In general, phytobiotic additives improve growth performance and milk yield by increasing energy utilization efficiency in the rumen, reducing energy loss in the form of methane, and redirecting it to milk and meat production (Cohen-Zinder et al. 2016). Meanwhile, PSMs, especially tannin and flavonoids, improve N utilization by decreasing protein degradation in the rumen and increasing bypass protein in the small intestine, resulting in better ruminant performance (Herremans et al. 2020). Furthermore, phytobiotic additives promoted ruminal bacteria associated with meat and milk production (Li et al. 2020). For example, Hassan et al. (2020) reported that buffaloes were fed a mixture of phytogenic substance-promoted bacteria that have been correlated with milk and fat yield (e.g., Firmicutes-to-Bacteroidetes ratio, *Pseudobutyrivibrio*, *Butyrivibrio*, and *Succinivibrioanceae*).

The properties of antiprotozoa agents of PSMs, such as tannin and saponin, increase microbial protein biomass and thus increase microbial protein bypass to the intestine, promoting amino acid absorption in the gut (Abarghuei and Salem 2021). Furthermore, phytobiotic additives improve animal metabolic status by controlling the secretion of various endocrine hormones via the hypothalamus-pituitary axis, such as prolactin and growth hormone, and modulating the insulin/like growth factor-I(IGF-1) signaling pathway, which is related to better lactation performance, as reported in a study by Li et al. (2020) when buffaloes were fed mulberry leaf flavonoids.

## Effects of phytobiotic additives on product quality

In recent years, research has focused on reducing saturated fatty acids (SFA) and increasing n-3 polyunsaturated fatty acid (n-3 PUFA), conjugated linolenic acid (CLA) in animal products such as milk and meat (Shingfield et al. 2013). The presence of n-3 PUFA and CLA in animal products benefits humans by preventing a variety of disorders and diseases (e.g., muscular degeneration, asthma, psychiatric disorders, hypertension, psychiatric disorders, cardiovascular diseases, antiatherosclerosis, antidiabetic, anticancerogenic, and antiobesity), as reviewed by (Lin et al. 2016).

Previous research has shown that phytobiotic additives alter the fatty acid profiles of animal products, which is associated with improved human health outcomes (Makmur et al. 2022). For example, feeding *Acacia cyanophylla* leaves to dairy ewes reduced oleic acid while increasing minor (n-6) fatty acids such as linolenic acid (C18:3 cis6 cis9 cis12 (n-6)) and docosapentaenoic acid (Maamouri et al. 2019). According to Huang et al. (2022), feeding ensiled paulownia leaves to dairy cows increased proportions of* α*-linolenic acid, CLA, C18:1 trans-11 fatty acid, PUFA, and reduced n6/n3 ratio and SFA proportion in milk.

On meat fatty acid profiles and quality, Arend et al. (2022) found that finishing cattle fed on 58% grape pomace had high content *Longissimus lumborum* and semimembranosus muscle of fatty acids such as 18:2 n-6, 18:2 c9t11, CLA, and PUFA and reduced lipid oxidation. Uushona et al. (2023a) indicated that the inclusion *A. mearnsii* leaf-meal at 200 g/kg DM in lamb finisher diets enhanced meat fatty acid composition by reducing individual and total SFA and increased rans (t)-monounsaturated fatty acid (MUFA) mainly t10/t11–18:1, individual and total CLA, n-3 and n-6 PUFA contents as well as improved meat antioxidant capacity, lightness, oxymyoglobin content and decreased deoxymyoglobin content, lipid oxidation and shear force.

Several factors could explain the alteration of fatty acid profiles in milk and meat caused by phytobiotic additives. First is the modification of the ruminal biohydrogenation process, specifically the inhibition of the final step in the biohydrogenation of vaccenic acid to stearic acid (Khiaosa-Ard et al. 2009). For instance, Emami et al. (2015) observed a linear increase in vaccenic acid, CLA, and punicic acid concentrations in subcutaneous and intramuscular fat with increasing pomegranate seed pulp levels in the diet of kids. More recently, Birkinshaw et al. (2022) reported that tannin-containing feeds, such as vine leaves, lowered the n-6:n-3 fatty acid ratio and increased concentrations of vaccenic and rumenic acids in the milk of dairy cows. On the contrary, according to Baila et al. (2023), phytobiotic additives inhibit ruminal biohydrogenation in the early stages, indicating that lactating ewes fed sainfoin proanthocyanidins had higher milk PUFA contents and a decrease in MUFA intermediates such as vaccenic acid. Further, Dias Junior et al. (2023) observed that essential oil from *Arnica montana* decreased linearly the C17:0, C18:0, C18:1 trans-11, C22:6 n3, and the sum of SFA, and linearly increased the C18:2 cis-9, cis-12; C18:3 cis-9, cis-12, cis-15, the sum of PUFA, and the sum of n6 in the meat of lambs.

Several factors, including the dose and chemical structure of botanical compounds (Patra and Saxena 2011), interactions between diet ingredients (Vasta et al. 2009), and possibly between-animal variability Harnly et al. (2022), may explain the variation between phytobiotic additives inhibiting ruminal biohydrogenation in several stages. Second, phytobiotic additives alter the composition of rumen microbes and metabolic pathways, resulting in the accumulation of PUFA and CLA in the products (Denninger et al. 2020). For example, adding mulberry leaf silage to lamb diets increases the content of unsaturated fatty acids in the longissimus dorsi muscle by increasing the relative abundance of *Christensenellaceae* (R-7), *Bifidobacterium*, and *Lactobacillus* in the rumen, which has a positive correlation with n-3 PUFA, CLA, and eicosapentaenoic acid in ruminant products, according to Xiong et al. (2021) and Wang and Luo (2021).

## Effects of phytobiotic additives on animal health

As a natural alternative to anthelmintic drugs, phytobiotic additives have been used to treat gastrointestinal parasitism (Busari et al. 2021; Pech-Cervantes et al. 2021). This is because drug residues can pass into products (e.g., meat and milk), which might negatively impact humans and make worm populations resistant to anthelmintics (Sutherland and Leathwick 2011).

Numerous studies have been accomplished on the anthelmintic properties of phytobiotic additives, whether they are whole plants, active components, or plant extracts (Alowanou et al. 2019; Tchetan et al. 2022). Various plant extracts (*Artemisia campestris*, *Salix caprea*, and *Punica granatum*) have been used to treat gastrointestinal nematodes (GIN) in lambs, according to Castagna et al. (2021). The results showed that a *P. granatum*-based remedy reduced GIN egg output by 50%. Furthermore, according to Pelegrin-Valls et al. (2022), feeding suckling lambs sainfoin has a positive effect on reducing coccidian oocysts. Phytobiotic additives, particularly those with high polyphenol content (e.g., tannins and flavonoids), triterpenoids, and saponin, have anthelmintic effects by forming complexes with protein in the rumen and increasing amino acid absorption by the small intestine (Tchetan et al. 2022), which improves host homeostasis and immunomodulatory of the host against various parasites (Min et al. 2003). Given their protein-binding capability, it appears probable that tannins possess a broad-spectrum action rather than targeting specific components, enabling them to effectively combat various structures within nematodes (Greiffer et al. 2022). In addition to their nematicidal effects, tannins also demonstrate other anthelmintic activities, such as the inhibition of egg hatch, suppression of larval motility, and prevention of larval exsheathment (Spiegler et al. 2017). Saponins have potential applications in controlling internal parasites in ruminants (Kholif 2023). They possess inhibitory effects on proteases, lipases, and chitinases, enzymes responsible for degrading egg membranes crucial for nematode egg hatching. Disruption of these enzyme activities interferes with the hatching process, leading to the elimination of infectious worms (Botura et al. 2013).

Inflammation, immunodeficiency, and oxidative stress have all been shown to harm farm animals, particularly during the transitional period in dairy cows, when changes in endocrine and metabolic status are required to prepare for parturition and lactogenesis (Sordillo and Aitken 2009). There is an imbalance between the production of oxidants (radicals and non-radicals) and their detoxification by the antioxidant system during this period, which impairs immune responses and causes diseases (Halliwell 2007). Furthermore, rumen non-adaptation to starch-rich diets or insufficient dry matter intake for the animal, as well as heat stress during this period, will expose the animal to metabolic disorders such as rumen acidosis and hyperketonemia, which are all factors contributing to high oxidative stress (Guo et al. 2013).

Phytobiotic additives have antioxidant and immune-enhancing properties that reduce oxidative stress, lipid peroxidation, and the inflammatory response in Holstein steers fed on botanical blends such as micro-encapsulated cinnamon and oregano essential oils, free turmeric extract, and tannic acid, as demonstrated by Brunetto et al. (2023). According to Safari et al. (2018), feeding dairy cows with pomegranate seed pulp during the postpartum period enhanced antioxidant status, which was related to a decrease in lipid oxidation (free fatty acids and β-hydroxybutyrate) and malondialdehyde (MDA) as well as an increase in superoxide dismutase activity, hence preventing cells from oxidative stress. In a study by Vizzotto et al. (2021), it was found that feeding Jersey cows oregano extract at a rate of 10 g/cow/day during prepartum and postpartum reduced the levels of reactive oxygen species (ROS) in the erythrocytes by 40% and that feeding green tea extract at a rate of 5 g/cow/day reduced the levels of reactive species during prepartum and postpartum by 24 and 29%, respectively. In dairy cows with hyperketonemia, Ma et al. (2021) found that supplementing with green tea polyphenols from 15 days prepartum to 30 days postpartum reduced somatic cells count and improved antioxidative status by lowering concentrations of oxidative stress biomarkers like ROS, hydrogen peroxide, and MDA while promoted concentrations of interleukin-6 and interleukin-10 and diminished concentrations of tumor necrosis factor-α, interleukin-1β, interleukin-2, interleukin-8, and interferon-ϒ in plasma.

Phytobiotic additives’ antioxidant properties are explained by increasing the activity of antioxidant enzymes that eliminate free radicals and decreasing the accumulation of MDA and ROS by activating the NFE2L2/heme oxygenase-1 (HMOX1) pathway, which improves cell growth and metabolism (Ma et al. 2019). Also, phytobiotic additives modulate the inflammatory response by increasing anti-inflammatory cytokine concentrations and decreasing pro-inflammatory cytokine concentrations in plasma, which protects host tissue from damage (Ma et al. 2021).

Furthermore, the modulation of ruminal and hindgut microbiota that interacts with host metabolism and physiology may explain the increase in animal antioxidant capacity or immunomodulatory effects of phytobiotic additives (Xie et al. 2020). According to Wang and Luo (2021), lambs fed mulberry leaf silage had higher levels of *Bifidobacterium*, *Lactobacillus*, and *Schwartzia*. The authors of the previous study have established that the presence of *Schwartzia* bacteria is positively correlated with antioxidant function; this is due to competition with methanogenic bacteria, whereas *Bifidobacterium* and *Lactobacillus* are associated with a highly positive correlation with serum IFN-ϒ, which is involved in the initiation and regulation of the immune response. Furthermore, citrus-derived flavonoids with high flavanones and O-polymethoxylated flavones improve dairy cow inflammatory status by promoting hindgut fermentation and increasing probiotics *Bacteroides*, *Phascolarctobacterium*, *Bifidobacterium* spp., and *F. prausnitzii*, while decreasing *Clostridium cluster* XIVab, *E. coli*, and *Ruminococcus torques* group according to Zhao et al. (2023).

## The combination of phytobiotic additives with other feed additives

In recent years, researchers have looked into the potential benefits of the synergistic effect of phytobiotic additives and probiotics in the livestock industry to achieve the best growth performance and health status. Despite this, there are very few papers that report the synergistic effect of two additives, with the majority of studies focusing on claves. For example, Seifzadeh et al. (2017) found that combining a medical plant mix with probiotics did not improve clave growth performance. On the other hand, Liu et al. (2020) found that adding essential oils and prebiotics to starter feed at 44 mg/calf/day improved calf growth, ruminal development, gut health, nutrient digestibility, and immunity. Additionally, Stefańska et al. (2021) observed that combining 50 mg of rosmarinic acid per calf per day with a multi-strain Lactobacillus probiotic (250 mg per calf per day) during the preweaning period in neonatal calves had antiparasite effects, increased ruminal VFA, bacteria, and protozoa, and increased blood insulin-IGF-1 and β-hydroxybutyrate. The disparity between studies was most likely caused by differences in dose, the bacterial strain composition of the probiotics, and the chemical structure and concentrations of the bioactive compounds in the herbal extracts, as well as different ration compositions and animal management strategies, such as a milk replacer feeding model (Schären et al. 2017; Uyeno et al. 2015). In young ruminants, probiotics have been suggested to improve intestinal health by increasing mucosal immunity and preventing the proliferation of pathogenic bacteria (Ayyat et al. 2023) by producing a variety of antimicrobial compounds such as bacteriocins, hydrogen peroxide, VFA, and nitric oxide, allowing the probiotic bacteria to compete with other gut bacteria (native or pathogenic species) and induce the equilibrium between intestinal microorganisms and promote rumen fermentation (Sun et al. 2016; Uyeno et al. 2015). Meanwhile, phytobiotic additives had antimicrobial, anti-inflammatory, antioxidant activities and endocrine stimulants (Kumar et al. 2022; Zhao et al. 2023), all highlighted a positive synergistic effect between phytobiotic additives and probiotics. However, further studies are needed to investigate measures of ruminal fermentation and rumen development when young ruminants are fed a combination of probiotics and phytobiotics as well as their impacts on animal productivity and measure CH_4_ emission in adult ruminants (Jia et al. 2022).

## Safety and regulatory considerations for phytobiotic additives in ruminant diets

Although phytobiotic additives are rich in valuable compounds that can improve animal health, productivity, and feed efficiency, their use in animal feed requires careful consideration of safety and regulatory requirements to ensure the health and welfare of the animals as well as the safety of the resulting products. One of the main safety considerations for phytobiotic additives in ruminant diets is the risk of toxicity. Despite having positive health effects at low concentrations, the presence of bioactive components like tannin, saponin, alkaloids, cardiac glucosides, and cyanogenetic glucosides can have detrimental effects on animals when consumed in large quantities (An et al. 2023; Costa et al. 2021; Mickdam et al. 2016; Seyedin et al. 2023). Therefore, it is important to carefully select phytobiotic additives that have been shown to be safe and effective in ruminant diets. The use of phytobiotic additives should be based on scientific evidence and should follow recommended dosage guidelines. Also, safety considerations should require residue to determine whether any harmful residues from phytobiotic additives remain in animal products such as meat or milk (Franz et al. 2020).

Another safety consideration for phytobiotic additives is the risk of contamination with harmful substances, such as heavy metals, pesticides, and mycotoxins. The use of contaminated phytobiotic additives in animal feed can have negative impacts on animal health and product safety (Franz et al. 2020). Therefore, it is important to source phytobiotic additives from reputable suppliers and to test them for contaminants before use. Regulatory considerations for phytobiotic additives in ruminant diets include compliance with feed safety regulations and labeling requirements. In many countries, the use of phytobiotic additives in animal feed is regulated by government agencies, such as the Food and Drug Administration in the United States or the European Food Safety Authority in the European Union. These agencies set standards for feed safety and require that phytobiotic additives be labeled accurately and clearly (EFSA 2020). The labeling requirements may include accurate ingredient listing, dosage instructions, storage conditions, withdrawal periods (if applicable), and any cautionary statements regarding potential side effects or contraindications.

Other factors to consider include the metabolization (Kim et al. 2021) or adaptation (Benchaar et al. 2008) of microbiota to phytobiotic additives over time by the formation of an extracellular polysaccharide coat and the formation of the electro-dense layer commonly seen at the cell surface of bacteria (Smith et al. 2005). Because of the variety of active component types and chemical profiles, bacteria may lack the ability to develop protective mechanisms against each compound (López et al. 2010). As a result, long-term studies on phytobiotic additives are needed to determine the start of the bioactive effect as well as adaptation to the natural additives.

## Conclusion and future directions

Phytobiotic additives are safe and effective alternatives to antibiotics in animal feed, with benefits for both animal nutrition and the environment. These additives reduce nitrogen excretion and methane emissions from animals, protecting the environment. They also manipulate rumen fermentation, increasing ruminant productivity by maintaining a higher ruminal pH, reducing ruminal protein degradation, and increasing energy. Phytobiotic additives have additional benefits, including anthelmintic, antioxidative, and anti-inflammatory immunomodulatory activity, improving animal product quality and human health. However, further research is needed to understand the effects of phytobiotic additives on the microbiome, the PSM conversion rate and final products of native PSM, and the mechanisms by which probiotics and botanical additive formulation inhibit ruminal methanogenesis and nitrogen utilization in dairy cows. Similarly, more studies are required to uncover the effects of citrus flavonoid intake on hindgut fermentation, microbiome, and metabolites in dairy cows, as well as the regulatory mechanisms involved in the metabolic health effects of phytobiotic additives and their metabolites in the gastrointestinal tract of dairy cows.

## Data Availability

Not applicable.
